# Planning and process evaluation of a multi-faceted influenza vaccination implementation strategy for health care workers in acute health care settings

**DOI:** 10.1186/1471-2334-13-235

**Published:** 2013-05-23

**Authors:** Josien Riphagen-Dalhuisen, Gerard Frijstein, Nannet van der Geest-Blankert, Marita Danhof-Pont, Herbert de Jager, Nita Bos, Ed Smeets, Marjan de Vries, Pieter Gallee, Eelko Hak

**Affiliations:** 1 Department of PharmacoEpidemiology & PharmacoEconomics, University Centre of Pharmacy, University of Groningen, A. Deusinglaan 1, P.O. Box XB45, Groningen 9713 AV, the Netherlands; 2 Department of Epidemiology, University Medical Centre Groningen, Groningen, the Netherlands; 3 Department of Occupational Health and Environment, Academic Medical Centre, Amsterdam, the Netherlands; 4 Department of Occupational Health and Environment, University Medical Centre St. Radboud Nijmegen, Nijmegen, the Netherlands; 5 Department of Occupational Health and Environment, Leiden University Medical Centre, Leiden, the Netherlands; 6 Department of Occupational Health and Environment, Erasmus Medical Centre Rotterdam, Rotterdam, the Netherlands; 7 Department of Occupational Health and Environment, University Medical Centre Utrecht, Utrecht, the Netherlands; 8 Department of Medical Microbiology, Maastricht University Medical Centre, Maastricht, the Netherlands; 9 Department of Occupational Health and Environment, University Medical Centre Groningen, Groningen, the Netherlands; 10 Department of Occupational Health and Environment, Free University Medical Centre, Amsterdam, the Netherlands

**Keywords:** Influenza vaccination, Health care workers, Intervention mapping, Intervention implementation, Acute health care

## Abstract

**Background:**

Influenza transmitted by health care workers (HCWs) is a potential threat to frail patients in acute health care settings. Therefore, immunizing HCWs against influenza should receive high priority. Despite recommendations of the World Health Organization, vaccine coverage of HCWs remains low in all European countries. This study explores the use of intervention strategies and methods to improve influenza vaccination rates among HCWs in an acute care setting.

**Methods:**

The Intervention Mapping (IM) method was used to systematically develop and implement an intervention strategy aimed at changing influenza vaccination behaviour among HCWs in Dutch University Medical Centres (UMCs). Carried out during the influenza seasons 2009/2010 and 2010/2011, the interventions were then qualitatively and quantitatively evaluated by way of feedback from participating UMCs and the completion of a web-based staff questionnaire in the following spring of each season.

**Results:**

The IM method resulted in the development of a transparent influenza vaccination intervention implementation strategy. The intervention strategy was offered to six Dutch UMCs in a randomized in a clustered Randomized Controlled Trial (RCT), where three UMCs were chosen for intervention, and three UMCs acted as controls. A further two UMCs elected to have the intervention. The qualitative process evaluation showed that HCWs at four of the five intervention UMCs were responsive to the majority of the 11 relevant behavioural determinants resulting from the needs assessment in their intervention strategy compared with only one of three control UMCs. The quantitative evaluation among a sample of HCWs revealed that of all the developed communication materials, HCWs reported the posters as the most noticeable.

**Conclusions:**

Our study demonstrates that it is possible to develop a structured implementation strategy for increasing the rate of influenza vaccination by HCWs in acute health care settings. The evaluation also showed that it is impossible to expose all HCWs to all intervention methods (which would have been the best case scenario). Further study is needed to (1) improve HCW exposure to intervention methods; (2) determine the effect of such interventions on vaccine uptake among HCWs; and (3) assess the impact on clinical outcomes among patients when such interventions are enacted.

## Background

Influenza is an annual respiratory infection which has the capacity to cause severe morbidity and mortality, particularly among frail hospitalized patients. The influenza attack rate among health care workers (HCWs) can be considerable [1], with studies showing that more than 75% continue to work after infection [2,3]. As HCWs can transmit influenza to their patients, immunizing them against influenza is an extremely important measure to protect patients from the viral infection [4,5]. Such vaccination has proven to be effective in preventing influenza infection among HCWs themselves since they are generally young and able to mount a more effective immune response when compared to frail patients [6]. In a recent systematic review, Osterholm et al. found a significant pooled influenza vaccine efficacy with an estimated reduction in influenza of 59% among young adults [7]. Whilst the number of available studies is limited, influenza vaccination has also been shown to reduce influenza-like illness-related absenteeism of HCWs [8], which is essential to preserve continuity of care. Using a micro-simulation hospital department model, Van den Dool et al. demonstrated that, although no herd immunity can be achieved, there is an inverse linear relationship between the number of vaccinated HCWs and the number of infected hospital patients, meaning that each additional HCW who is immunized against influenza adds to the preventive effect [9]. These clinical trial studies demonstrating the effects of immunizing HCWs against influenza on patient outcomes were all conducted in long-term care facilities [10], and it should be noted that acute care hospital settings are very different compared to long-term care as they have a higher patient turnover, which hampers the applicability of findings from long-term care settings to acute care settings [9].

Following guidelines set by the World Health Organisation, the Dutch Health Council has (as of 2007) recommended influenza vaccination for HCWs in contact with high-risk patients in the Netherlands, but vaccine coverage of HCWs has been low. For example, in 2006 and 2008 in all eight Dutch University Medical Centers (UMCs) vaccination uptake among HCWs ranged from 0% to 28%, with an average uptake of 13%. Such low vaccine coverage appeared to be consistent with European figures reported in a study by Blank et al., which showed low influenza vaccine coverage of HCWs in 11 European countries, with a maximum coverage of a low 26% in the Czech Republic [11].

Using special interventions, it is possible to increase influenza vaccine coverage of HCWs in acute health care settings. In a before-after trial from Spain, Llupia et al. demonstrated an increase in vaccine coverage of HCWs from 23% in 2007/2008 to 37% in 2008/2009 by means of a promotional and educational strategy [12], but they did not report a systematic method for developing their strategy. In the Netherlands, Looijmans-van den Akker et al. developed a systematic program to increase vaccine uptake among HCWs in nursing homes. After the intervention, the influenza vaccine uptake in the intervention group was on average 9% higher than in the control group (p = 0.02). However, it should be noted that the applicability of these findings to acute care settings is likely to be limited [13]. To extract the full value of an influenza vaccination strategy in hospitals, a theoretical framework that underpins the development of such a strategy is essential, especially for future applications. For the study reported in this paper, we have used the Intervention Mapping (IM) method to systematically plan, develop and evaluate the process of an influenza vaccination implementation strategy [14]. To the best of our knowledge, our study is the first report on the development of an implementation strategy that targets influenza vaccine uptake among HCWs in acute care settings which includes a process evaluation. The effects of the developed intervention program on actual behaviour, and the clinical outcomes, will be separately reported as part of a cluster randomized controlled trial.

## Methods

### Setting and trial design

This report outlines the development of the intervention and process evaluation as part of an intervention trial conducted in the Netherlands during the seasons 2009/2010 and 2010/2011 [trial number NCT01481467]. With the permission from the board of directors, and with permission from the Dutch Association of UMCs (Nederlandse Federatie van Universitair Medische Centra), all eight Dutch UMCs participated in the study. Six UMCs agreed to be randomized to receive either the intervention (3 UMCs) or act as controls (3 UMCs), and two UMCs chose to implement the developed intervention program (the ‘external intervention UMCs’). Formal ethical approval to conduct the implementation trial, according to the Dutch Law of Research with Humans, was not required (Medical Ethical Committee, University Medical Center Groningen, Netherlands, No. 2009.267). The study was conducted in accordance with the Dutch Law for the Protection of Personal Data (Wet Bescherming Persoonsgegevens), and the Declaration of Helsinki [http://www.wma.net/en/30publications/10policies/b3/].

**Figure 1:**
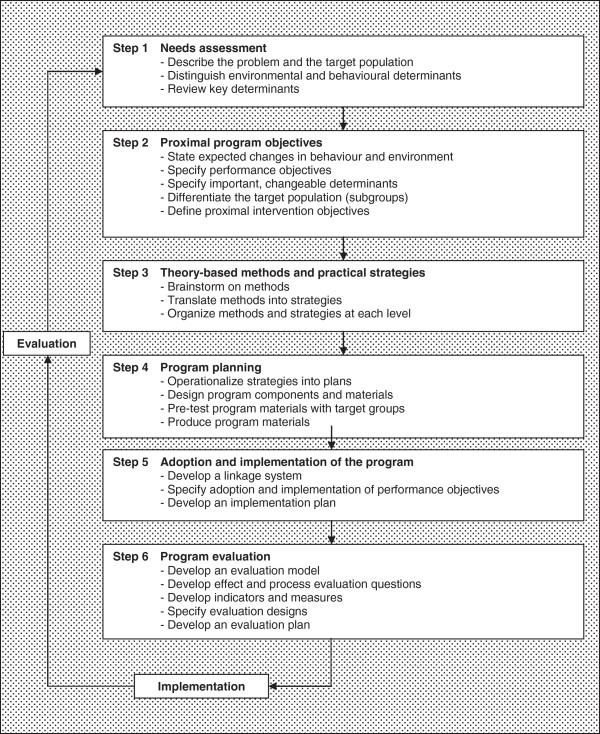
**Intervention mapping method (adapted from Bartholomew et al.)**[14]**.**

The Intervention Mapping (IM) method [14] was used to develop, implement and evaluate the process of the intervention strategy for HCWs. The IM method is a framework for systematically developing health education interventions, and can be used as part of the dynamic process of planning intervention strategies in health education. The process of developing and evaluating an implementation strategy is composed of six steps: 1) a needs assessment; 2) establishment of proximal program objectives; 3) development of theory-based methods and practical strategies; 4) program planning; 5) adoption and implementation of the program; and 6) program evaluation (see Figure 1).

### Developing the program according to the IM method

#### Step one: Needs assessment

To gain insight into how to improve the influenza vaccine coverage of HCWs, we first assessed the relevant determinants of influenza vaccination behaviour. In 2008, prior to the onset of the 2009 trial, a questionnaire-based study was performed among HCWs of five selected departments from the group of eight participating University Medical Centres (UMCs) [15]. Based on the Health Belief Model and the Behavioural Intention Model demographical, behavioural and organisational determinants were assessed [16,17]. Multivariate analysis of the responses resulted in an 11-item prediction model, with two relevant demographic and nine behavioural determinants (the results of which are presented in Table 1). The final prediction model showed a high discriminative value (area under the receiver operating curve: 0.95), meaning that on the basis of the presence or absence of these determinants, vaccination behaviour of 95% of HCWs can be accurately predicted.

**Table 1:** Determinants associated with influenza vaccination uptake among health care workers (HCWs) resulting from the needs assessment

**Determinants**	**Odds ratio**	**Changeability**^**a**^	**Category**	**Target group**^**b**^
Demographic				
Age >40 years	2.65	-	Not applicable	Not applicable
Chronic illness	3.37	-	Not applicable	Not applicable
Behavioural				
Aware of personal risk for influenza infection	2.80	+	Knowledge	2
Aware of risk of infecting patients	2.54	+	Knowledge	2
‘Vaccination reduces risk of infecting patients’	3.68	+	Knowledge	2
‘Vaccination is useful despite the constant flow of visitors’	1.88	+	Knowledge	2
Aware of the contents of the Health Council’s Advice	2.41	+	Knowledge	2
‘HCWs^c^ should get vaccinated to ensure continuity of care’	2.15	+	Common interest	3
‘HCWs should get vaccinated because of their duty to do no harm’	2.22	+	Common interest	3
‘People around me think it is important for me to get vaccinated’	1.74	+/-	Social impact	3
‘I would definitively get vaccinated if it was available at a convenient time’	28.91	+	Organizational	1,2,3

^a^ - : not changeable, + : changeable as discussed in our 10-person research team under supervision of a communication expert.

^b^ Target group 1: HCWs who deliberately comply with vaccination.

Target group 2: HCWs who deliberately do not comply with vaccination.

Target group 3: HCWs who unintentionally do not comply with vaccination.

^c^ HCWs: health care workers.

#### Step two: Proximal program objectives

Each of the 11 determinants associated with influenza vaccination compliance were discussed by our 10-person research team (the principal researchers/authors of this study) in order to determine which behavioural determinants could reasonably be changed through an implementation strategy. Decisions were taken by consensus, using an independent facilitator with expertise in the area of influenza vaccination from the National Institute of Health and the Environment (Bilthoven, the Netherlands). For these discussions, the core research team of the ten principal researchers was expanded by inclusion of the UMC research contacts (physicians from the departments of Occupational Health and Environment, or from the departments of Microbiology) who were in charge of the planning and implementation of the annual influenza vaccination strategy in their hospitals. Based on the measures of association (odds ratios) obtained from the 2008 questionnaire study [15], and in order to demonstrate the independent relevance of the determinants for potential change in behaviour (Table 1), the discussion group divided the determinants into different categories so as to target the use of methods/materials. The following categories were identified: knowledge; common interest; social impact; and organizational (see Table 1).

One of the critical assessments in developing an implementation strategy for changing behaviour is exploring whether the person’s behaviour is intentional or not. The research team identified three different target groups among HCWs: (1) HCWs who deliberately choose to comply; (2) HCWs who deliberately choose not to comply; and (3) those HCWs who unintentionally do not comply with vaccination. The varying methods/materials are separated according to target groups in the IM matrix, but in best practice all three target groups were exposed to all developed methods in line with the proximal objectives (see Table 2).

**Table 2:** Proximal program objectives and methods

**Determinants**	**Proximal program objectives**	**Methods/materials**
*Demographic*		
Age >40 years	Not applicable due to limited changeability	- Not applicable
Chronic illness	Not applicable due to limited changeability	- Not applicable
*Behavioural*		
Aware of personal risk for influenza infection	Create awareness among HCWs of the risk to get infected with influenza and it’s consequences	- Provide information on influenza, transmission and risks through an information stand at the UMC restaurants, a website, a folder and plenary meetings
		- Polls and a quiz on the intranet
		- Video testimonials with role models
Aware of risk of infecting patients	Create awareness among HCWs of the risk to transmit influenza to patients and how vaccinating HCWs can prevent this	- Provide information on influenza and the risk of transmission to patients through an information stand at the UMC restaurants, a website, a folder and plenary meetings
		- Polls and a quiz on the intranet
		- Video testimonials with role models
‘Vaccination reduces risk of infecting patients’	HCWs being convinced that vaccinating HCWs against influenza will reduce the risk of transmission to patients	- Provide information on influenza and the effectiveness of vaccination through an information stand at the UMC restaurants, a website, a folder and plenary meetings
		- Polls and a quiz on the intranet
		- Video testimonials with role models
‘Vaccination is useful despite the constant flow of visitors’	HCWs being convinced that vaccinating HCWs is useful despite the constant flow of visitors	- Provide information on influenza and the effectiveness of vaccination through an information stand at the UMC restaurants, a website, a folder and plenary meetings
		- Polls and a quiz on the intranet
		- Video testimonials with role models
Aware of the contents of the Health Council’s Advice	Create awareness among HCWs on the existence and contents of the guideline developed by the Dutch Health Council	- Provide and explain contents of the advice on the intranet or website
		- Explain and discuss in a plenary meeting
‘HCWs should get vaccinated to ensure continuity of care’	HCWs understand the ethical aspects of this matter and the need to ensure continuity of care	- Explain and discuss ethical aspects (plenary meeting, website)
		- Video testimonials with role models
		- Involve Board of Directors (e.g. first vaccination, be present at vaccination, column)
		- Distribute badges to vaccinated HCWs saying ‘deliberately vaccinated for you’ to start the discussion
‘HCWs should get vaccinated because of their duty to do no harm’	HCWs understand the ethical aspects of vaccinating HCWs and that this is part of their duty of care	- Explain and discuss ethical aspects (plenary meeting, website)
		- Video testimonials with role models
		- Involve Board of Directors (e.g. first vaccination, be present at vaccination, column)
		- Distribute badges to vaccinated HCWs saying ‘deliberately vaccinated for you’ to start the discussion
‘People around me think it is important for me to get vaccinated’	Create awareness of the importance of vaccination among those close to the HCWs	- Personal invitation letter with information folder and a link to the website at the home address
‘I would definitively get vaccinated if it was available at a convenient time’	Create a more convenient approach	- Poster with clear practical information on location and time
		- Personal invitation at home address with location and time
		- Extended vaccination hours which take changing shifts into account

#### Step three: Theory-based methods and practical strategies

To influence the behaviour of a target group, a wide range of intervention methods/materials is required and these need be propagated through different channels and means [14]. Bartholomew et al., for example, provides theoretical methods for major behavioural determinants as well as for all higher environmental levels [14]. After reviewing the literature pertaining to vaccine studies [2,18-21] the research team agreed on the methods to be implemented. As no simple practical strategies or methods exist that guarantee success [22], we took the different target groups into account when developing the tools. Examples of methods at the individual level included: participation in information meetings; consciousness raising by way of letters of invitation for vaccination; persuasive communication (such as a dedicated website with clear messages); interactive learning through ‘frequently asked questions’ or polls on a website; tailored to different target groups. HCWs who intentionally do not comply with influenza vaccination, need to be provided with clear information in order to eliminate any possible misconceptions or misunderstandings (e.g. on absence of vaccine effects or risk of serious adverse effects) so that they may change their views. In contrast, for HCWs who unintentionally remain unvaccinated it is more important to increase their awareness of their behaviour and its possible consequences. Testimonials from role models (e.g. members of the Board of Directors or Heads of Departments),where the reasons to comply with the vaccination program are provided, can play an important role in this awareness change. Thus, by actively promoting the vaccination campaign, and by demonstrating the importance of vaccination in a variety of ways, vaccine coverage of HCWs was expected to be improved.

#### Step four: Program planning

The topics and channels of the strategy methods were discussed individually by the lead investigator with members of the research team. After a number of meetings, consensus was reached in each UMC about the program methods to be used, and the best way to design and produce them. Common formats and sample materials were developed and pre-tested by the research team, which were subsequently adapted by the communication departments of the individual UMCs. A dedicated website, http://www.bewustgepriktvooru.nl (in Dutch), was developed by a web designer using the structure and contents produced by the research team. The Dutch Federation of UMCs (the NFU) and the Dutch association of nurses and nursing assistants (the V&VN) indicated their support by their approval for their logos to be displayed on the website. In order to stimulate discussion among HCWs, badges were developed with the tagline “bewust geprikt voor u” (Dutch for ‘deliberately vaccinated for you’), to be handed out to HCWs after vaccination. The badges were designed by an external designer in two forms, one for HCWs working on regular wards, and a child-friendly badge for HCWs working on the paediatric ward (showing a hedgehog). In support of the intervention, the research team also provided written information about the relevance of influenza vaccination for HCWs and about the time and location of vaccination, for use on individual hospital intranet websites and/or in folders and leaflets. To engage HCW staff in the project, a quiz was also developed that was made available on the project website. The effective exchange and availability of these developed materials to members of the research team, and the contact persons of the intervention UMCs was facilitated by making them accessible on a secured section of the project website.

#### Step five: Adoption and implementation of the program

To achieve the highest impact, the implementation of the developed strategy needed to be arranged in a programmatic and structured fashion. As a first step, the intervention UMC contacts and relevant communication staff were visited by the communication expert to explain and discuss the timelines and program of the implementation strategy before and during the vaccination campaign. For further assistance, all UMC contact persons were able to pose questions, or to initiate discussions on the secured section of the central project website. During the vaccination campaign members of the research team were also available for questions and advice. The team also developed news items for use by UMC communication officers to raise awareness among HCWs. In line with current practice, all intervention UMCs were free to choose the methods that were most appropriate to them. The three control UMCs were asked to carry out their own annual influenza vaccination program as planned, without putting more efforts into their strategy than normal, and without using any of the intervention program materials and/or strategies that were developed by the research team.

#### Step six: Program evaluation

Both a qualitative and quantitative process evaluation was carried out. Part of the qualitative process evaluation was conducted through the completion of set checklists by the contact person from each intervention UMC. In addition, annual communication reports on the influenza vaccination campaign were compiled by the communication offices of all UMCs, providing summaries of the evaluation of the intervention program by the teams involved in the organisation of the influenza vaccination program. In addition, UMC contacts were invited to comment on the methods/materials used in the intervention campaign. The checklists and reports were then reviewed for the number of behavioural determinants that the actual implementation strategy at each of the UMCs targeted. These are presented as a ‘yes/no’ per determinant (see Table 3).

**Table 3:** Evaluation of the use of behavioural determinants in vaccination campaign by implementers of the intervention UMCs (N is given)

**Determinants**	**Intervention UMCs**	**External intervention UMCs**	**Control UMCs**
	**n = 3**	**n = 2**	**n = 3**
Aware of personal risk for influenza infection	2/3	2/2	2/3
Aware of risk of infecting patients	3/3	2/2	3/3
‘Vaccination reduces risk of infecting patients’	2/3	2/2	2/3
‘Vaccination is useful despite the constant flow of visitors’	2/3	1/2	1/3
Aware of the contents of the Health Council’s Advice	3/3	2/2	1/3
‘HCWs^a^ should get vaccinated to ensure continuity of care’	2/3	2/2	1/3
‘HCWs should get vaccinated because of their duty to do no harm’	2/3	2/2	2/3
‘People around me think it is important for me to get vaccinated’	1/3	1/2	1/3
‘I would definitively get vaccinated if it was available at a convenient time’	3/3	2/2	1/3

^a^ HCWs: health care workers.

To obtain more detailed quantitative information on the process variables, in both intervention and control UMCs, we developed a web-based questionnaire for HCWs of the five selected departments that were also involved in the 2008 questionnaire study by Hopman et al. (two intensive care units, internal medicine, paediatric ward and neonatology) [15]. An email invitation with a link to the web-based questionnaire was sent to the heads of the five departments after both influenza study seasons, requesting them to invite their HCW staff to complete the questionnaire. The study participants included nurses, physicians and support staff. The questionnaire assessed vaccination determinants as well as possible exposure to the developed materials, e.g. folders, posters, the website and testimonials, and how these were rated (e.g. ‘have you noticed posters in your UMC’; ‘did you like them’; rated on a 5-point Likert scale).

## Results

### Results of the process evaluation

#### Qualitative process evaluation in the intervention and control UMCs

Table 3 shows the qualitative evaluation of the methods that were applied in both intervention and control UMCs. Though the intervention program focused on the specific determinants according to the study of Hopman et al. [15], the control UMCs might independently also have focused their program on one or more of these determinants. With the exception of the determinants “Vaccination is useful despite the constant flow of visitors” and “People around me think it is important for me to get vaccinated”, the determinants (c.f. Table 1) were targeted by four or all five intervention UMCs, compared with fewer by the control UMCs. Both intervention and control UMCs targeted the determinant “awareness of risk”.

From the communication reports derived from the intervention UMCs, it became evident that (1) longer opening hours for administration of the vaccine, (2) more vaccination locations, and (3) the use of mobile carts appeared to be associated with an increased vaccine uptake among HCWs. Providing information on influenza and vaccination by different means (intranet, posters, magazine and letters) was found to be very useful. Although there was not much difference in the level of involvement of the Boards of Directors of the intervention UMCs compared with the control UMCs, the self-reported impression by the UMC evaluation teams was that such involvement led to positive intentions among HCWs. Two intervention UMCs organized plenary and interactive meetings for HCWs where information on influenza, the influenza vaccination and the determinants was provided, and where HCWs were given the opportunity to ask questions. In contrast, in the communication reports of the control UMCs it was stated that the information provided to staff was too limited, and with only one control UMC organizing a plenary information meeting.

#### Quantitative evaluation of the implementation process in the intervention group

In the quantitative evaluation, a sample of HCWs from five selected departments of the participating UMCs was asked to complete an anonymous web-based questionnaire. In the spring of 2010, 2,255 HCWs were approached, of whom 678 (249 from intervention UMCs) completed the questionnaire (response rate of 30.1%). In the spring of 2011, 4,885 HCWs were invited to participate in the questionnaire with 908 (303 from intervention UMCs) responses (response rate of 18.6%). Baseline data of participants were similar across study seasons and UMCs. Respondents were predominantly female (in 2009/2010 88.9% in the ‘external intervention group’ and 86.7% in the ‘intervention group’, *p* = 0.554). The proportion of HCWs older than 45 years was similar across seasons and groups, ranging from 37.8% to 42.7%. More nursing staff than physicians participated in the questionnaire (nursing staff ranging from 86.4% to 99.2%), and overall response rates varied by department, with the highest response rates in the paediatric ward and the lowest response rates in the internal medicine department.

Table 4 summarizes the questionnaire results from the intervention UMCs across study seasons concerning the usage of the developed tools in their UMC. As the findings for the three ‘intervention UMCs’ and the two ‘external intervention UMCs’ were similar, the results for both sets of UMCs were combined. In the pandemic influenza season of 2009/2010, approximately 25% of HCWs attended an information meeting on influenza. One year later, approximately 10% of HCWs attended such an information meeting. In the pandemic season (2009/2010) the badges were handed out to around 32.9% of HCWs, while in the 2010/2011 season this number was almost halved (16.6%). In addition, a higher proportion of the handed-out badges was worn in the pandemic season than in the 2010/2011 influenza season. Of all the developed communication materials, HCWs reported the posters as the most noticeable.

**Table 4:** Quantitative evaluation: percentage of health care workers (HCWs) within intervention UMCS during study year 2009/2010 and 2010/2011 that used and appreciated the methods/materials

**Methods/materials**	**Intervention UMCs**	**Intervention UMCs**
	**2009/2010**	**2010/2011**
	**n = 249 HCWs**	**n = 303 HCWs**
	**(%)**	**(%)**
Visited the website	9.6	19.7
Attended information meeting	4.1	9.0
Badge was handed out	32.9	16.6
Wore the badge	20.5	14.3
Rated the badge as appealing	3.2	7.4
Rated the poster(s) as appealing	9.6	7.9*
Rated the folder as appealing	9.2	3.3*
Rated the video(s) as appealing	2.8	1.3

*P < 0.05.

## Discussion

In this study we have demonstrated how the IM method by Bartholomew et al. [14] can be applied to develop a structured immunization strategy to increase the influenza vaccine coverage of HCWs in acute care settings. According to the process evaluation we were able to implement such a strategy in participating hospitals. Compared with the Dutch study performed by Looijmans-van den Akker et al. in nursing homes, our IM-based intervention achieved an increased attendance rate of HCWs at information meetings of 24% in the ‘pandemic’ 2009/2010 influenza season, and 9% in the ‘normal’ 2010/2011 influenza season, when compared with the observed 7% participation rate in the nursing home study [13]. Our evaluation showed that posters were an efficient tool for use in acute care settings as these were most commonly noticed by the HCWs. However, it appeared to be impossible to achieve a 100% exposure of every HCW to all materials, which would be the best case scenario.

At the core of this implementation study was the systematic planning of the program and the selection of methods according to the IM method, in consultation with a communication expert. Using a number of discussion sessions the team agreed upon and developed different methods/materials to be directed at the different target groups. The inclusion of an assessment of the needs of each intervention UMC enhanced the program’s applicability. The diversity of backgrounds of the research team members (ranging from physicians to hospital hygienists) was considered an advantage since this led to a wider perspective during the development of the different implementation tools.

A possible limitation to the current study may be the observed discrepancy between the findings of the qualitative and quantitative evaluation. In the qualitative evaluation, most of the three ‘intervention’ and the two ‘external intervention’ UMCs reported that the majority of the nine behavioural determinants were taken into account, and that most of the proposed methods were implemented. However, the quantitative questionnaire results showed that the actual exposure of HCWs to these developed tools appeared suboptimal. This discrepancy may in part be due to the lower response rates to the web-based questionnaire, notably during the second study season. Although the response rate in the first season was rated ‘quite high’ for such evaluations and ‘acceptable’ during the second season, bias may have occurred such that respondents were more negative (or positive) regarding the program than the average HCW. Since we did not pursue a non-responder study, the direction of such potential bias remains undetermined. In the nursing home study by Looijmans et al. [13], a clear trend towards higher vaccine coverage of HCWs was observed when nursing homes implemented more components of the intervention program. Therefore, whilst it is clearly difficult to achieve full exposure to the different program elements, future programs should consider exposure to all intervention program elements as part of their aim of achieving optimal influenza vaccine coverage [22].

Another possible limitation of this study was the widespread pandemic of new influenza A(H1N1) that occurred during the study period. Our evaluation showed that during an influenza pandemic methods/materials were used and rated differently when compared with the normal (seasonal) influenza period. For instance, more HCWs attended information meetings on influenza and vaccination in the pandemic season than during the normal season. It should also be noted that the influenza pandemic caused a lot of anxiety and media attention in the Netherlands and in the participating hospitals. In particular, it was predicted that many hospital admissions could be expected as well as understaffing of hospitals by HCWs’ absenteeism. As a consequence, extra efforts were made towards vaccinating HCWs against new influenza A(H1N1). Although this external effect will have interfered with the purpose and conduct of the randomized intervention trial in the pandemic year, the increased attention was national and can be assumed to have been similar for both intervention and control UMCs. Therefore the conclusions from our study based on relative performance of the intervention and control UMCs should still be valid.

## Conclusions

A structured implementation strategy for promoting influenza vaccination amongst HCWs was developed using the IM method and trialled over two influenza seasons in 5 UMCs. A process evaluation showed that the intervention could be successfully implemented in acute health care settings. Whilst the evaluation showed increased vaccination uptake by HCW staff of the participating UMCs, it also showed that it was impossible to expose all HCWs to all intervention methods (which would be the best case scenario). Further study is needed to (1) improve HCW exposure to intervention methods; (2) evaluate the effect of such interventions on vaccine uptake among HCWs; and (3) assess the impact on clinical outcomes among patients in hospitals where such interventions are enacted.

## Competing interests

All authors declare that they have no competing interests.

## Authors’ contributions

JRD conducted the study, collected and analyzed the data and drafted the manuscript. GF, NGB, MDP, HJ, NB, ES, MV and PG all contributed to the design of the study, were lead contacts during the study and critically reviewed the manuscript. The authors also formed the 10-person research team that discussed the determinants listed in Table 1. EH obtained funding, supervised the conduct and report of the study and critically commented on the manuscript. All authors read and approved the final version of a manuscript.

## Pre-publication history

The pre-publication history for this paper can be accessed here:

http://www.biomedcentral.com/1471-2334/13/235/prepub
